# Design of hepadnavirus core protein-based chimeric virus-like particles carrying epitopes from respiratory syncytial virus

**DOI:** 10.1038/s41541-024-00855-7

**Published:** 2024-03-19

**Authors:** Shuai Shao, Xue Feng Zhang, Jun Wei Hou, Sen Sen Yang, Zi Bo Han, Hai Lan Wu, Fang Tang, Xin Yu Li, Ze Hua Lei, Zi Xin Zhao, Shu Xiang Li, Zhao Ming Liu, Pu Shan, Yu Qin Jin, Ji Guo Su, Yu Liang, Jing Zhang, Qi Ming Li

**Affiliations:** 1 The Sixth Laboratory, National Vaccine and Serum Institute (NVSI), Beijing, China; 2 National Engineering Center for New Vaccine Research, Beijing, China; 3 The Third Laboratory, National Vaccine and Serum Institute (NVSI), Beijing, China; 4 High Performance Computing Center, National Vaccine and Serum Institute (NVSI), Beijing, China

**Keywords:** Protein vaccines, Protein vaccines

## Abstract

Respiratory syncytial virus (RSV) is one of the most important pathogens causing respiratory tract infection in humans, especially in infants and the elderly. The identification and structural resolution of the potent neutralizing epitopes on RSV fusion (F) protein enable an “epitope-focused” vaccine design. However, the display of RSV F epitope II on the surface of the widely-used human hepatitis B virus core antigen (HBcAg) has failed to induce neutralizing antibody response in mice. Here, we used the hepadnavirus core protein (HcAg) from different mammalian hosts as scaffolds to construct chimeric virus-like particles (VLPs) presenting the RSV F epitope II. Mouse immunization showed that different HcAg-based chimeric VLPs elicited significantly different neutralizing antibody responses, among which the HcAg derived from roundleaf bat (RBHcAg) is the most immunogenic. Furthermore, RBHcAg was used as the scaffold platform to present multiple RSV F epitopes, and the immunogenicity was further improved in comparison to that displaying a single epitope II. The designed RBHcAg-based multiple-epitope-presenting VLP formulated with MF59-like adjuvant elicited a potent and balanced Th1/Th2 immune response, and offered substantial protection in mice against the challenge of live RSV A2 virus. The designed chimeric VLPs may serve as the potential starting point for developing epitope-focused vaccines against RSV. Our study also demonstrated that RBHcAg is an effective VLP carrier for presenting foreign epitopes, providing a promising platform for epitope-focused vaccine design.

## Introduction

Respiratory syncytial virus (RSV) is a highly contagious pathogen that can cause severe lower respiratory tract infections in infants, young children, immunocompromised individuals, and the elderly. It has been reported that about 90% of children have experienced one or more RSV infections by two years of age^1^. RSV is an enveloped, negative sense, single-stranded RNA virus. Two glycoproteins on the surface of the RSV virion, i.e., the fusion (F) and attachment (G) proteins, play important roles in mediating the infection of host cells^2,3^. The G protein has high genetic diversity, whereas the F protein is more conserved across variant strains. The F protein is the main target for vaccine development^2,3^.

RSV vaccine development was plagued for a long time by the occurrence of vaccine-enhanced disease (VED) induced by formalin-inactivated virus^4,5^. In recent years, the crystal structures of both the pre-fusion (Pre-F) and post-fusion (Post-F) states provided structural insights into the conformation-dependent antigenicity of the RSV F protein^6,7^. Pre-F contains several immunodominant epitopes that disappear upon the spontaneous irreversible conformational transition from the Pre-F to the Post-F states. Pre-F can elicit a much higher level of neutralizing antibody response than Post-F, and therefore several vaccine candidates have been developed via stabilizing the F protein in its Pre-F state^8–12^. The Pre-F-based vaccines have achieved encouraging results in their clinical studies, and the vaccines developed by the pharmaceutical companies GSK and Pfizer have been approved by the U.S. Food and Drug Administration (FDA) for use to protect against RSV disease^13^.

Many neutralizing antibodies (nAbs) targeting RSV F protein have been isolated, several of which have been or are being optimized to develop therapeutics for clinical use. The monoclonal nAb palivizumab is available for high-risk infants to prevent severe disease caused by RSV infection^14^. Structural analysis has revealed that the antigenic site targeted by palivizumab is a helix–turn–helix motif, called epitope II, present both in the Pre-F and Post-F conformations. Recently, nirsevimab, a monoclonal nAb targeting the Pre-F-specific epitope Ø, was shown to be highly protective against RSV disease in several clinical trials in infants and has been approved by the European Medicines Agency (EMA) and U.S. FDA (https://www.fda.gov/news-events/press-announcements/fda-approves-new-drug-prevent-rsv-babies-and-toddlers) as a broadly protective option against RSV for infants^15–17^.

Progress in the structural analysis of the F protein-nAb complexes as well as the antigenic site responsible for the binding of nAbs has enabled the vaccine design focusing on the neutralizing epitopes. So far, at least seven neutralizing epitopes, i.e., Ø, I, II, III, IV, V and VIII, have been identified. Epitopes I, II and IV are present in both the Pre-F and Post-F conformations, whereas epitopes Ø, III, V and VIII are specific to the Pre-F state^2,18–21^. Antigenic site II is the target of the nAbs palivizumab and motavizumab, and palivizumab has been used clinically to prevent RSV disease for many years. Epitope VIII is bound by nAb hRSV90 isolated from human blood samples^20,21^. Both sites II and VIII consist of a linear region of the F protein primary sequence, which facilitates the rational vaccine design by displaying these epitopes on immunogenic carriers, such as virus-like particles (VLPs).

Human hepatitis B virus (HBV) core antigen (HBcAg) has been widely used as an antigen-display system for VLP-type vaccine design. It is highly immunogenic because of its repetitive presentation of foreign antigenic epitopes^22^. Correia et al. designed a stable structure containing the epitope II from RSV F protein, which was then conjugated repeatedly to the HBcAg particles. However, the constructed chimeric VLP failed to induce neutralizing response in mice^23^. Interestingly, Schickli and colleagues utilized the hepadnavirus core protein from woodchuck (WHcAg) to display the RSV F protein epitope II, and this chimeric VLP induced high-titer neutralizing antibodies both in mice and rats^24^. These results indicate that the epitope-presenting scaffold also plays an important role in effectively eliciting a neutralizing antibody response. Hepadnavirus has also been reported in, but not limited to, ground squirrel (GSHV), arctic ground squirrel (AGSHV), bat (BTHV) and roundleaf bat (RBHV)^25^. In this study, the core proteins from these different hepadnaviruses, i.e., WHcAg, GSHcAg, AGSHcAg, BTHcAg and RBHcAg, were used as scaffolds to present epitope II of the RSV F protein, and the neutralizing antibodies induced by these various chimeric VLPs in mice were evaluated and compared. Our results show that the VLPs from different species displaying epitope II elicited obviously varied neutralizing antibody levels in mice, and the RBHcAg was found to be the most immunogenic scaffold for the presentation of the RSV epitope II.

Furthermore, extensive studies have revealed that several positions in HBcAg protein, including the major immunodominant region (MIR) at the tips of the virion surface, the N-terminus and the C-terminus, can accommodate large foreign sequences without hindering particle assembly^22,26–29^. Using the RBHcAg, which is structurally homologous to HBcAg, as the scaffold platform^30^, antigenic sites II and VIII of RSV F protein were inserted into different regions of the scaffold to construct various chimeric VLPs harboring multiple RSV epitopes. Mouse immunization tests show that the RBHcAg-based chimeric VLPs carrying one RSV epitope II in the MIR and two epitopes VIII on the N- and C-termini produced a higher neutralizing response than that only harboring one epitope II on the MIR. The designed multiple-epitope-presenting VLP formulated with MF59-like adjuvant elicited a potent and balanced Th1/Th2 immune response in mice, and offered substantial protection against live RSV A2 virus challenge. Our results demonstrated that RBHcAg can serve as an effective VLP carrier to present neutralizing epitopes from RSV F protein, providing a potentially promising platform for the epitope-focused vaccine design.

## Results

### Construction and characterization of chimeric VLPs presenting RSV F protein epitope II on different species of HcAg as the scaffold

**Fig. 1:**
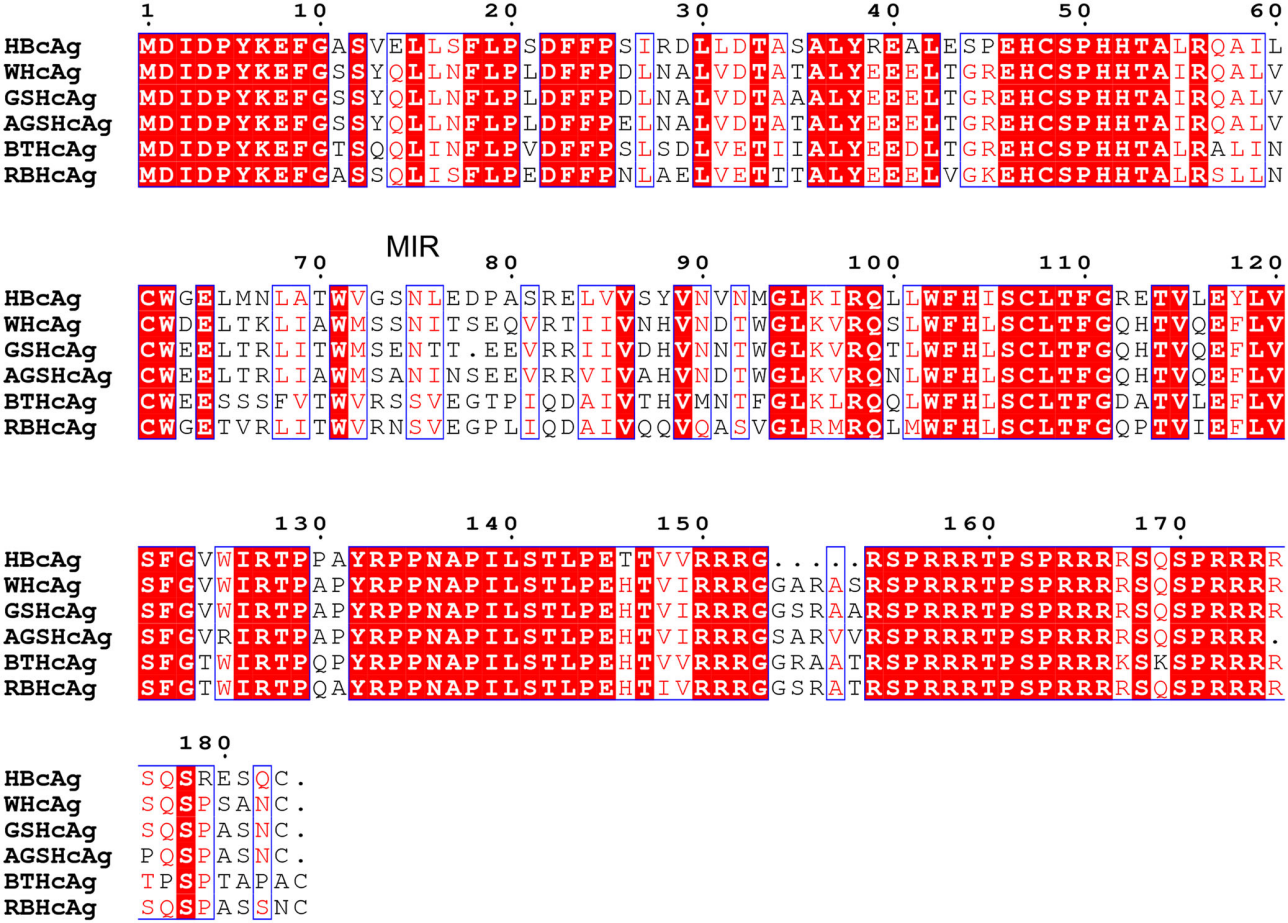
Sequence alignment of the HBcAg, WHcAg, GSHcAg, AGSHcAg, BTHcAg and RBHcAg. The residue numbering was based on the sequence of HBcAg.

Epitope II (a.a. Asn254-Asn277) of the RSV F protein is a potent neutralizing antigenic site recognized by palivizumab, a licensed therapeutic for RSV infection. It serves as a potential target for epitope-focused vaccine design. Virus-like particles (VLPs) are an ideal platform to improve immunogenicity through repeated presentation of multiple copies of the epitopes on a nano-size particle. In this study, the RSV epitope II was fused to the major immunodominant region (MIR) of the hepadnavirus core protein (HcAg) to construct chimeric VLPs. To investigate the immunological effects of different VLP carriers, six HcAgs derived from varied species, including HBcAg, WHcAg, GSHcAg, AGSHcAg, BTHcAg and RBHcAg, were each used as scaffolds to display the RSV F epitope II. Sequence alignments by ENDscript^31^ show that the percentages of identity between these HcAgs are in the range of 66–92%. The MIR is one of the most diverged regions (Fig. 1). The differences in the immunogenicity between these different chimeric VLPs constructed with these various HcAgs were compared in this study. In addition, the studies of the Milich group have shown that adding short linkers to the ends of the inserted epitope favored better presenting the antigenic conformation of the epitope^24,32^. Therefore, the RSV F epitope II with two short connecting linkers were inserted at residues 78/79 on the tip of HcAg MIR to construct the hybrid VLPs. Based on the above design schemes, a total of 6 chimeric VLPs harboring RSV F epitope II were constructed, as listed in Table 1, and the immunogenicity of these different constructs was evaluated.

**Table 1:** Design of chimeric VLPs harboring RSV F protein epitope II

Design scheme	VLP carrier	Linker	Inserted epitope	Linker
HBcAg_MIR-II	HBcAg	GILA	NSELLSLINDMPITNDQKKLMSNN	L
WHcAg_MIR-II	WHcAg	GILA	NSELLSLINDMPITNDQKKLMSNN	L
GSHcAg_MIR-II	GSHcAg	GILA	NSELLSLINDMPITNDQKKLMSNN	L
AGSHcAg_MIR-II	AGSHcAg	GILA	NSELLSLINDMPITNDQKKLMSNN	L
BTHcAg_MIR-II	BTHcAg	GILA	NSELLSLINDMPITNDQKKLMSNN	L
RBHcAg_MIR-II	RBHcAg	GILA	NSELLSLINDMPITNDQKKLMSNN	L

**Fig. 2:**
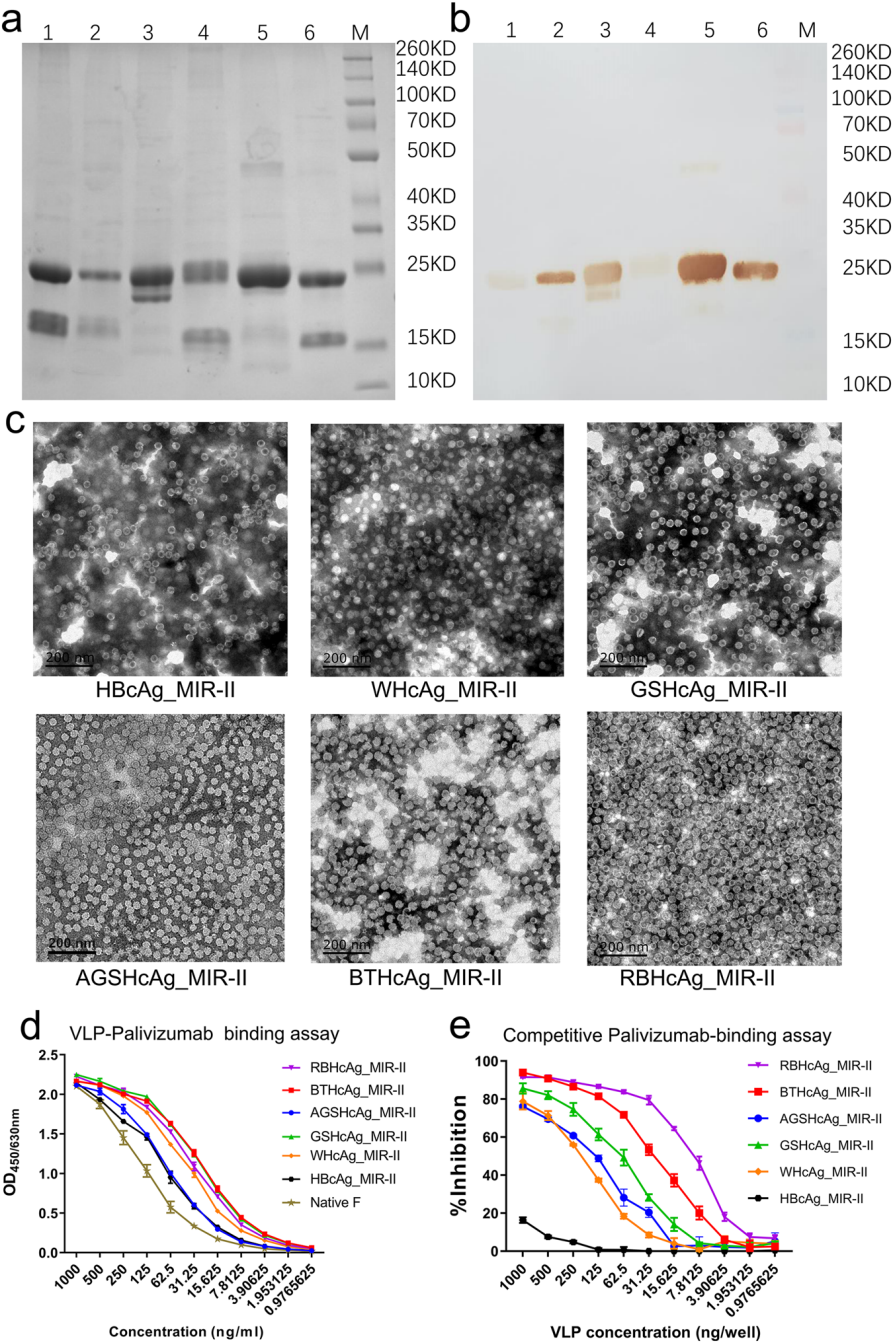
Recombinant expression, characterization, and bioactivity evaluation of the designed chimeric VLPs presenting RSV F epitope II by using different species of HcAg as the scaffolds. The designed chimeric VLPs were expressed by transfecting the *hansenula polymorpha* using a system developed by our laboratory. **a**, **b** SDS-PAGE (**a**) and Western-blot using palivizumab (**b**) for the designed chimeric VLPs, respectively. Lane 1: BTHcAg_MIR-II, Lane 2: AGSHcAg_MIR-II, Lane 3: WHcAg_MIR-II, Lane 4: HBcAg_MIR-II, Lane 5: GSHcAg_MIR-II, Lane 6: RBHcAg_MIR-II, Lane M: Protein molecular weight marker. **c** TEM microscopic images of the designed chimeric VLPs (scale bar 200 nm). **d** Concentration-dependent binding abilities of the designed chimeric VLPs to palivizumab tested using ELISA, which were compared with that of the native RSV F protein. Triplicate measurements were performed, and data are presented as mean ± SEM. **e** Competitive palivizumab-binding abilities of the designed chimeric VLPs in contrast with the native RSV F protein tested using ELISA. In this assay, the concentration of the native RSV F protein was 0.5 μg/ml (50 ng/well), and the VLP samples were serially diluted to different concentrations. Triplicate measurements were performed, and data are presented as mean ± SEM.

The designed chimeric proteins were expressed by the *hansenula polymorpha* yeast system developed by our laboratory^33,34^, and purified by gel filtration chromatography. SDS-PAGE analysis exhibited specific bands corresponding to the expected molecular weights of the chimeric proteins, indicating successful expression of all these six recombinant proteins, as shown in Fig. 2a and Supplementary Fig. 1a. The expression of the designed proteins was also verified by Western-Blot using palivizumab as the detection antibody, as shown in Fig. 2b and Supplementary Fig. 1b. To detect whether these chimeric proteins successfully self-assembled into VLPs, the morphology of the recombinant proteins was observed by using transmission electron microscope (TEM). TEM observation showed that all the designed proteins formed VLPs with uniform size and shape, as displayed in Fig. 2c, which are similar to the HBcAg particles reported by other groups^35,36^.

To determine whether the RSV F protein epitope II folded correctly and was also fully displayed on the scaffold, the binding abilities of the designed chimeric VLPs to the epitope-specific palivizumab were detected by using the enzyme-linked immunosorbent assay (ELISA). As a control, the binding strength of the native RSV F protein to palivizumab was also measured. The experimental results are displayed in Fig. 2d and Supplementary Table 1. All the designed chimeric VLPs exhibited high binding capability with palivizumab. Furthermore, competitive palivizumab-binding assays were also performed by using an ELISA to assess the relative binding affinities of the chimeric VLPs compared with the native F protein, as displayed in Fig. 2e and Supplementary Table 2. The results show that all the VLPs exhibited a reduction of palivizumab binding to the native RSV F protein. The extent of inhibition of the chimeric VLPs constructed based on the animal-derived HcAgs, including WHcAg, GSHcAg, AGSHcAg, BTHcAg and RBHcAg, was much higher than that of HBcAg. Among the designed chimeric VLPs, RBHcAg_MIR-II had the strongest inhibition capability, indicating the highest binding ability to plivizumab (Fig. 2e and Supplementary Table 2). The above results demonstrate that all these designed VLPs can present the RSV F epitope II, but the RBHcAg presents epitope II the most accurately and therefore is the most preferred scaffold.

### Immunological evaluation of different species of HcAg presenting RSV F protein epitope II

**Fig. 3:**
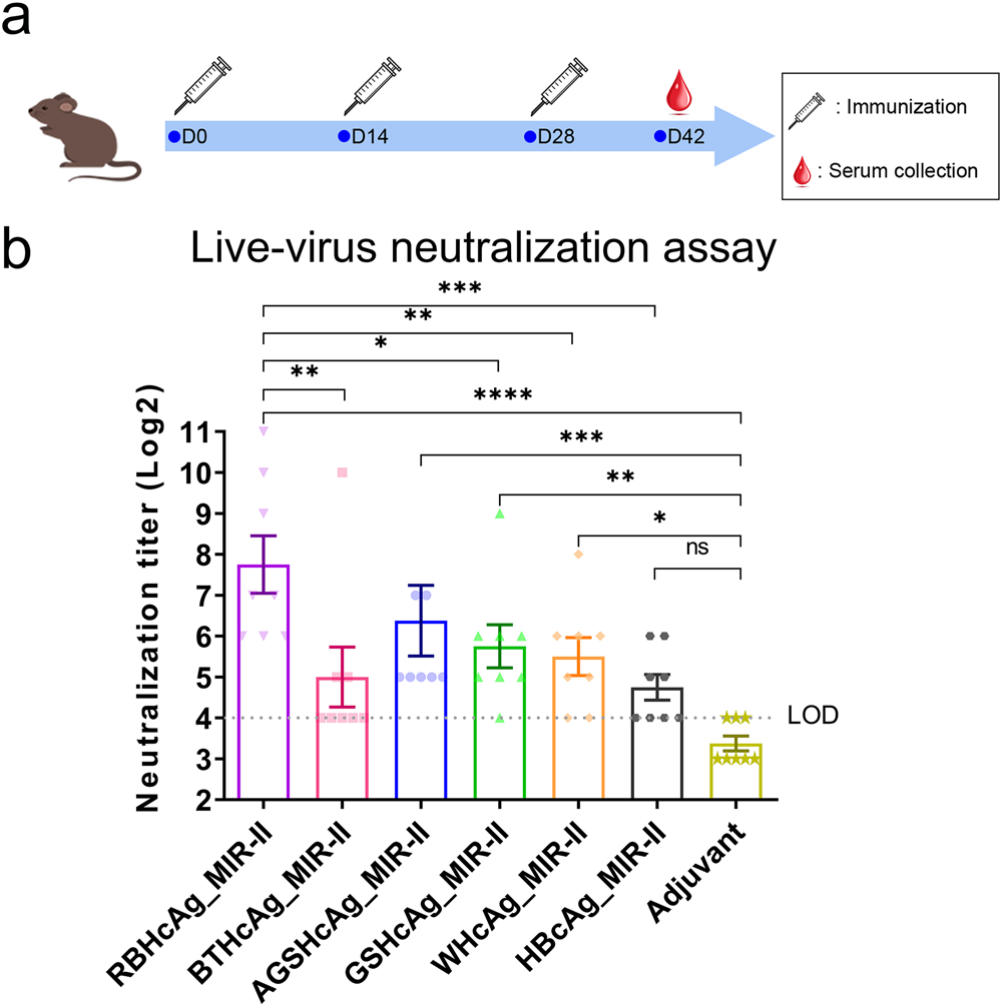
Comparison the immunogenicity of the designed different chimeric VLPs, including HBcAg_MIR-II, WHcAg_MIR-II, GSHcAg_MIR-II, AGSHcAg_MIR-II, BTHcAg_MIR-II, and RBHcAg_MIR-II. BALB/c mice (*n* = 8 per group) were immunized intraperitoneally on days 0, 14, and 28 with each chimeric VLP plus MF59-like adjuvant. Each dose contained 30 μg antigen (150 μl) and 150 μl adjuvant. Another group of mice was administered adjuvant only as a control. On day 14 after completion of the vaccination, neutralizing antibody titers in the serum of the immunized mice were measured using a live-virus neutralization assay. **a** Timeline of the mouse immunization and serum collection. **b** Live-virus neutralizing antibody titers in the serum of the mice immunized with different chimeric VLPs that display the RSV F epitope II. The quantification limit of the live-virus neutralization assay was 16, and the titer value below the limit of detection (LOD) was set to 8. Data are presented as mean ± SEM. The statistical significance of the difference between different groups was determined by One-way ANOVA with the LSD t-test. *p < 0.05, **p < 0.01, ***p < 0.001, ****p < 0.0001, ns not significant.

To evaluate the differences in the immunogenicity among the six designed chimeric VLPs that used different species of HcAg as scaffolds to display epitope II of the RSV F protein, mice were immunized by each chimeric VLP plus MF59-like adjuvant or only adjuvant, as a control. The neutralizing antibody titers in the sera of the immunized mice were measured by using a live-virus neutralization assay (Fig. 3a).

The neutralization assays show that the chimeric VLPs using different types of scaffold carriers induced various levels of neutralizing antibodies in mice (Fig. 3b and Supplementary Table 3). In the group immunized by the HBcAg_MIR-II construct, the neutralizing antibody titers for half of the mice were at the detection limit of the assay, and the geometric mean titer (GMT) was only 26.9. There was no statistical difference in the neutralizing antibody titers between the HBcAg_MIR-II and the adjuvant groups (Fig. 3b and Supplementary Table 3), which is consistent with previously reported results^23,24^. Whereas, the other chimeric VLPs that use the HcAgs from animals as scaffolds elicited relatively higher levels of neutralizing antibodies against RSV. The neutralizing titers induced by all the animal HcAg-based VLPs, except BTHcAg_MIR-II, were significantly higher than those of the adjuvant group. More importantly, the antibody titers elicited by RBHcAg_MIR-II were significantly higher than those of all the other chimeric VLPs except AGSHcAg_MIR-II (Fig. 3b and Supplementary Table 3). The geometric mean titers (GMTs) of the neutralizing antibodies reached 45.3, 53.8, 83.0, 32.0, and 215.3 for WHcAg_MIR-II, GSHcAg_MIR-II, AGSHcAg_MIR-II, BTHcAg_MIR-II, and RBHcAg_MIR-II, respectively. These results demonstrate that RBHcAg is the most immunogenic carrier for presenting the RSV F protein epitope II, which may serve as the preferred platform for epitope-focused vaccine design.

### Immunological evaluation of RBHcAg-based chimeric VLPs displaying multiple neutralizing epitopes from RSV F protein

Using the most immunogenic RBHcAg as the scaffold carrier, we then designed chimeric VLPs displaying multiple neutralizing epitopes from RSV F protein, and their immunogenicities were compared with the above constructed RBHcAg_MIR-II that only harbors one epitope of antigenic site II in the MIR. Similar to HBcAg, three regions in the RBHcAg, including the MIR, the N-terminus and the C-terminus, may be able to accommodate foreign epitopes. Therefore, based on the RBHcAg_MIR-II construct, the RSV antigenic site II or VIII (a.a. Glu163-Leu181) was further fused to the N- or/and C-terminus of RBHcAg. A total of four multiple-epitope-display VLPs were designed, in which epitope II was further inserted into the N-terminus (RBHcAg_MIR-II_N-II), C-terminus (RBHcAg_MIR-II_C-II), and both N- and C-termini (RBHcAg_MIR-II_NC-II) of RBHcAg_MIR-II, as well as epitope VIII was inserted into the N- and C-termini (RBHcAg_MIR-II_NC-VIII). The designed chimeric VLPs harboring multiple epitopes are listed in Table 2.

**Table 2:** Design of chimeric RBHcAg-based VLPs harboring multiple epitopes from RSV F protein

Design scheme	VLP carrier	N-terminal insert	MIR insert	C-terminal insert
RBHcAg_MIR-II_N-II	RBHcAg	NSELLSLINDMPITNDQKKLMSNN	**GILA**NSELLSLINDMPITNDQKKLMSNN**L**	none
RBHcAg_MIR-II_C-II	RBHcAg	none	**GILA**NSELLSLINDMPITNDQKKLMSNN**L**	NSELLSLINDMPITNDQKKLMSNN
RBHcAg_MIR-II_NC-II	RBHcAg	NSELLSLINDMPITNDQKKLMSNN	**GILA**NSELLSLINDMPITNDQKKLMSNN**L**	NSELLSLINDMPITNDQKKLMSNN
RBHcAg_MIR-II_NC-VIII	RBHcAg	EVNKIKSALLSTNKAVVSL	**GILA**NSELLSLINDMPITNDQKKLMSNN**L**	EVNKIKSALLSTNKAVVSL

The sequences highlighted in bold represent the linkers added to the ends of the epitope.

**Fig. 4:**
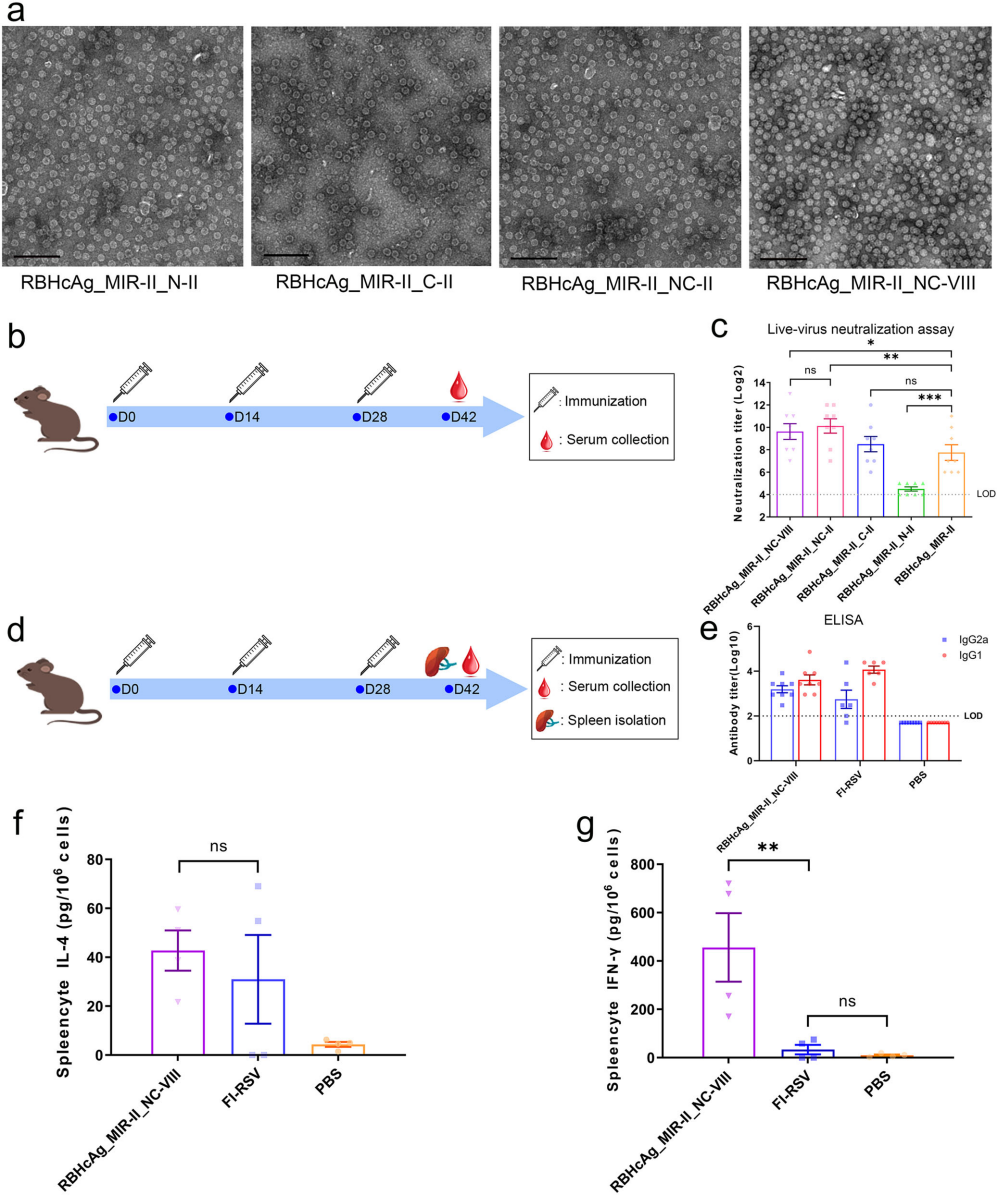
The designed RBHcAg_MIR-II_NC-VIII presenting RSV F epitope II in the MIR and epitope VIII in the N- and C-termini of RBHcAg induced not only high titers of neutralizing antibodies but also a balanced Th1/Th2 immune response. **a** Transmission electron microscopic (TEM) images (scale bar 200 nm) of the designed multiple-epitope-display VLPs, including RBHcAg_MIR-II_N-II, RBHcAg_MIR-II_C-II, RBHcAg_MIR-II_NC-II, and RBHcAg_MIR-II_NC-VIII. To evaluate the differences in the immunogenicity between the different chimeric VLPs harboring multiple RSV F neutralizing epitopes, BALB/c mice (*n* = 8 per group) were immunized intraperitoneally on days 0, 14, and 28 with each chimeric VLP plus MF59-like adjuvant. Each dose contained 30 μg antigen (150 μl) and 150 μl adjuvant. Another group of mice was administered the same amount of RBHcAg_MIR-II plus MF59-like adjuvant as a comparison. On day 14 after immunizing with the third dose, neutralizing antibody titers in the serum of the vaccinated mice were detected using live-virus neutralization assay. **b** Timeline of the mouse immunization and serum collection. **c** Neutralizing antibody titers in the mice elicited by different chimeric VLPs harboring multiple neutralizing epitopes, which were compared with those induced by the RBHcAg_MIR-II displaying only one epitope of antigenic site II. The quantification limit of the neutralization assay was 16. Data are presented as mean ± SEM. The statistical significance of the difference between different groups was determined by One-way ANOVA with the LSD t-test. *p < 0.05, **p < 0.01, ***p < 0.001, ns: not significant. To further evaluate the cellular immunity elicited by RBHcAg_MIR-II_NC-VIII and compare with that induced by FI-RSV, BALB/c mice (*n* = 8 per group) were immunized intraperitoneally with 30 μg (150 μl) RBHcAg_MIR-II_NC-VIII plus 150 μl MF59-like adjuvant, or with 2.5 × 10^5^ TCID_50_ FI-RSV plus 320 μg aluminum hydroxide, or with only PBS on days 0, 14, and 28. On day 14 after completion of the immunization, serum samples were collected and the RSV F-specific IgG1 and IgG2a antibody levels were detected using ELISA. In addition, the splenic lymphocytes of the immunized mice were isolated and stimulated using the live RSV A2 strain. The secretion of IL-4 and IFN-γ were quantified. **d** Timeline of the mouse immunization, serum collection, and spleen isolation. **e** The relative level of RSV F-specific IgG1 and IgG2a antibodies induced by RBHcAg_MIR-II_NC-VIII plus MF59-like adjuvant compared to that induced by FI-RSV. Two mice in the FI-RSV immunization group died and were removed from the analysis. The quantification limit of the assay was 100, and the titer value below the limit of detection (LOD) was set to 50. Data are presented as mean ± SEM. **f**, **g** The level of IL-4 (**f**) and IFN-γ (**g**) secretion in the splenic lymphocytes of the mice immunized with MF59-adjuvanted RBHcAg_MIR-II_NC-VIII, which was compared with that of mice immunized with FI-RSV. From each group, four mice were randomly selected, and the splenic lymphocytes were isolated. The secretions of IL-4 and IFN-γ by the splenocytes stimulated with RSV A2 virus were detected. Data are presented as mean ± SEM. Data was first tested for normal distribution using the Shapiro-Wilk test. For data with normal distribution, one-way ANOVA followed by the LSD t-test was used for multiple-group comparison. If the data did not follow a normal distribution, the Kruskal-Wallis test was used for multiple comparison. **p < 0.01, ns not significant.

The designed multiple-epitope-display VLPs were then expressed by using the *hansenula polymorpha* system and purified by gel filtration chromatography. TEM observations demonstrated that VLPs with uniform size and shape were formed for all chimeric proteins (Fig. 4a). To evaluate the immunogenicity of these multiple-epitope-display VLPs, mice were immunized with each VLP plus MF59-like adjuvant. The RBHcAg_MIR-II that harbors a single epitope II on its MIR was also used to immunize mice for comparison. Then, serum samples were collected from the immunized mice and neutralizing antibody levels were detected by the live-virus neutralization assay (Fig. 4b).

Mouse immunization experiments show that all the four chimeric VLPs presenting multiple epitopes elicited some level of neutralizing antibodies (Fig. 4c and Supplementary Table 4). The GMT of the neutralizing antibodies induced by RBHcAg_MIR-II_N-II was lower than that by RBHcAg_MIR-II, indicating that the insert of another epitope II into the N-terminus of RBHcAg_MIR-II did not further improve the neutralizing response. The neutralizing antibody GMT elicited by RBHcAg_MIR-II_C-II was comparable to that induced by RBHcAg_MIR-II. However, insertion of epitope II or VIII into both the N- and C-termini of RBHcAg_MIR-II dramatically improved the neutralizing antibody levels: the GMT value increased by nearly 4-fold and 3-fold for RBHcAg_MIR-II_NC-II and RBHcAg_MIR-II_NC-VIII, respectively (Fig. 4c and Supplementary Table 4). Taken together, among the four multiple-epitope-display VLPs, RBHcAg_MIR-II_NC-II and RBHcAg_MIR-II_NC-VIII stimulated the highest neutralizing antibody responses against RSV, which were significantly higher than that of the single-epitope-harboring RBHcAg_MIR-II. Because two different epitopes of the RSV F protein were displayed on RBHcAg_MIR-II_NC-VIII, this chimeric VLP was selected for further immunological investigations.

We then evaluated the cellular immunity elicited by the RBHcAg_MIR-II_NC-VIII, and compared with that induced by the formalin-inactivated RSV A2 strain (FI-RSV) adjuvanted with aluminum hydroxide. Two groups of mice were intraperitoneally injected with RBHcAg_MIR-II_NC-VIII plus MF59-like adjuvant or with the FI-RSV. Another group injected with phosphate buffer saline (PBS) as a control (Fig. 4d). Two mice in the FI-RSV immunization group died, which were removed from the analysis. The RSV F-specific IgG1 and IgG2a antibody levels in the serum of the immunized mice were detected by ELISA. The results show that the relative level of IgG1 and IgG2a antibodies induced by RBHcAg_MIR-II_NC-VIII formulated with MF59-like adjuvant was more balanced compared with those induced by the FI-RSV, indicating that more balanced Th1/Th2 cellular immunity was elicited by MF59-adjuvanted RBHcAg_MIR-II_NC-VIII than by the FI-RSV (Fig. 4e and Supplementary Table 5). Many previous studies have revealed that the imbalance in Th1/Th2 cytokine immune response is associated with the VED caused by the FI-RSV vaccine^37,38^. The more balanced Th1/Th2 antibody response may imply the alleviation or avoidance of VED for RBHcAg_MIR-II_NC-VIII plus MF59-like adjuvant as a vaccine candidate. The balanced Th1/Th2 immune response induced by MF59-adjuvanted RBHcAg_MIR-II_NC-VIII was also verified by the detection of the cytokine secretion levels. Four mice in each immunization group were randomly selected. The spleens of the selected mice were collected and the splenic lymphocytes were isolated. Then, the separated splenic lymphocytes were stimulated using the live RSV A2 virus, and the secretion levels of the virus-specific interleukin (IL)-4 and interferon (IFN)-γ were detected using the Luminex MAGPIX multiplex system. The detection results show that the IL-4 secretion level in the mice immunized with RBHcAg_MIR-II_NC-VIII was comparable to those immunized with FI-RSV, indicating that the similar level of Th2-type immune response was elicited between these two immunization groups (Fig. 4f and Supplementary Table 6). However, FI-RSV failed to elicit the secretion of IFN-γ, whereas a high secretion level was elicited by RBHcAg_MIR-II_NC-VIII. The IFN-γ secretion level in the RBHcAg_MIR-II_NC-VIII-immunized mice was significantly higher than that in the FI-RSV-immunized mice, which implies that much higher Th1-type immune response was induced by MF59-adjuvanted RBHcAg_MIR-II_NC-VIII compared with that by FI-RSV (Fig. 4g and Supplementary Table 7). Our results demonstrate that FI-RSV induced a Th2-biased response, whereas the designed RBHcAg_MIR-II_NC-VIII plus MF59-like adjuvant induce a balanced Th1/Th2 response, which is consistent with the above IgG1/IgG2a antibody detection results.

According to the above results of the animal experiments, the designed chimeric RBHcAg_MIR-II_NC-VIII formulated with MF59-like adjuvant elicited not only a potent but also a balanced Th1/Th2 immune response in mice.

### Protective effect provided by RBHcAg_MIR-II_NC-VIII in mice against challenge with live RSV A2 strain

**Fig. 5:**
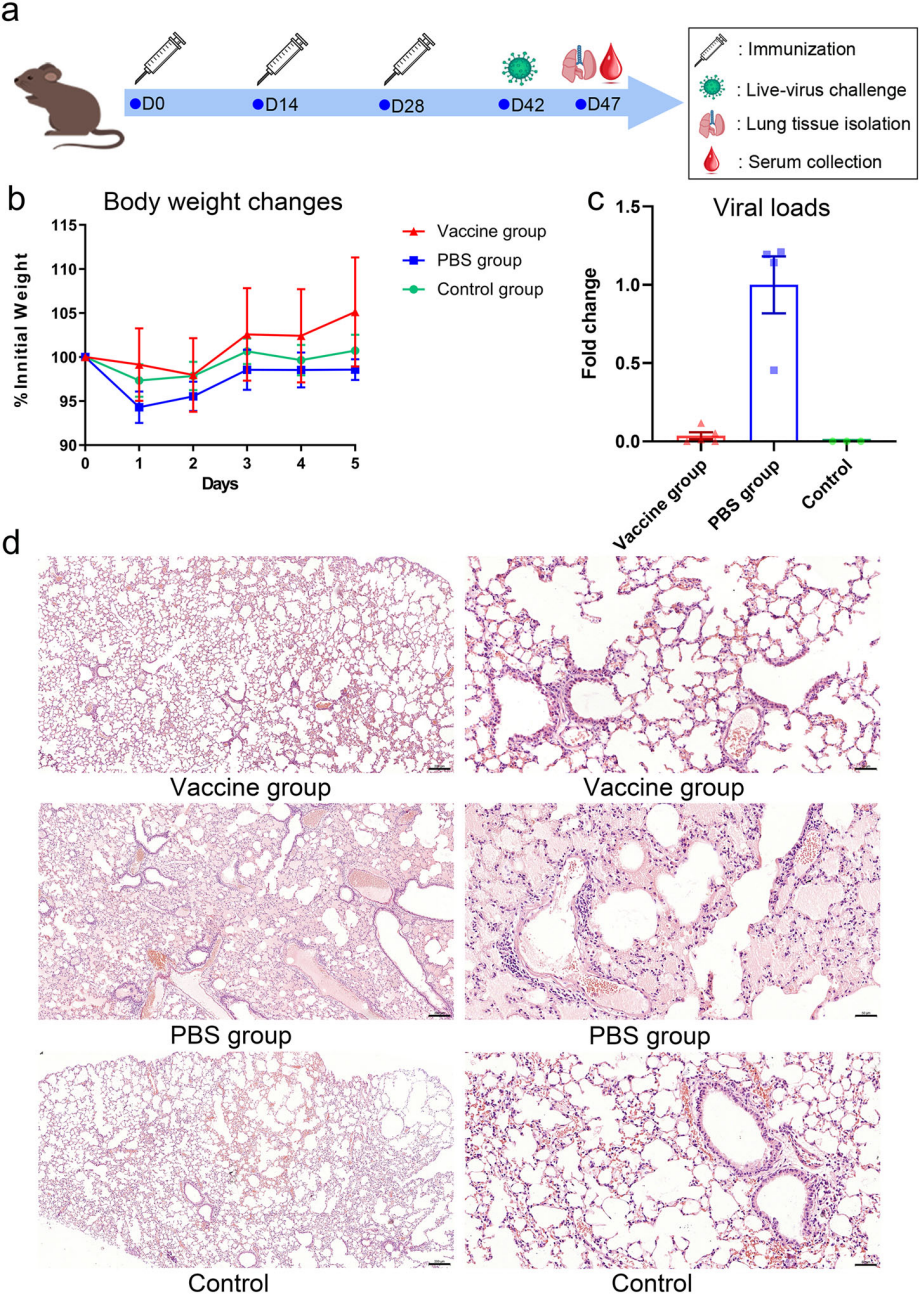
The designed RBHcAg_MIR-II_NC-VIII provided high protective efficacy in mice against challenge with live RSV A2 strain. BALB/c mice (*n* = 8 per group) were immunized intraperitoneally with RBHcAg_MIR-II_NC-VIII plus MF59-like adjuvant (vaccine group) or with PBS (PBS group) on days 0, 14, and 28, which were then challenged by RSV A2 strain on day 14 after the final immunization. Another group of mice (*n* = 4) was immunized and challenged with PBS as a control. Upon virus challenge, mouse body weights were measured. On day 5 after virus challenge, mice were sacrificed and their lung tissues were isolated, and then the virial loads in the lung tissues were detected and the histopathological changes were observed. **a** Timeline of mouse immunization, live-virus challenge, and lung tissue harvesting. **b** The body weight changes of the mice from different groups after virus challenge. One day after virus challenge, a mouse in the PBS group died and was removed from the analysis. Data are presented as mean ± SEM. **c** Comparison of the viral loads in the lung tissues of the mice from different groups. The viral load was tested using the reverse transcription real-time PCR, which was then analyzed by the comparative cycle threshold (Ct) method using the GAPDH gene as the calibrator. Detection results were reported as the fold changes of the viral loads in comparison to the mean value of the PBS group. Five, four, and three mice in the vaccine, PBS, and control groups, respectively, were used for viral load detections. Data are presented as mean ± SEM. **d** Histopathological images of the lung tissues from different groups of mice challenged with RSV A2 live virus. The remaining three, three, and one mouse in the vaccine, PBS, and control groups, respectively, were used for the histopathological examinations. Lef images: magnification 5× and scale bar 200 μm; Right images: magnification ×20 and scale bar 50 μm.

The protective efficacy of RBHcAg_MIR-II_NC-VIII was assessed in mice challenged with the live RSV A2 strain. Two groups of BALB/c mice were intraperitoneally immunized by three doses of RBHcAg_MIR-II_NC-VIII plus MF59-like adjuvant (vaccine group) or immunized by PBS (PBS group). Two weeks after completion of the immunization, the mice were challenged intranasally with the live RSV A2 strain. Another group of mice injected and challenged by PBS with the same regimen were used as a control. After virus challenge, the mice were weighted. Several previously reported studies have revealed that after the infection of RSV, the viral replication in the lung peaked at 4 to 6 days, and the pathological changes were most apparent between day 5 and 8 post-infection^39,40^. Therefore, on day 5 post challenge, the mice were sacrificed and the lung tissues of the mice were isolated (Fig. 5a). The viral loads and histopathological changes were examined. This time point has also been adopted for many other RSV challenge experiments in mice^41–43^.

One day after the virus challenge, a mouse in the PBS group died, which was removed from the analysis. Upon challenge with the RSV A2 virus, mice in the PBS group lost body weight. However, the body weight of the mice in the vaccine group increased (Fig. 5b and Supplementary Table 8). At the endpoint of the experiment, the mice were sacrificed, and the lung tissues were collected. The lung tissues of five, four, and three mice in the vaccine, PBS, and control groups, respectively, were used for viral load detections, and the remaining three, three, and one mouse in the three respective groups were used for histopathological examinations. High viral loads were detected in the mice from the PBS group, indicating severe RSV infections. In contrast, the viral loads for the mice in the vaccine group were similar to the control group, much lower than those in the PBS group. This result indicates effective protection was provided by the designed RBHcAg_MIR-II_NC-VIII vaccine in mice against the challenge of RSV (Fig. 5c and Supplementary Table 9). The protective efficacy of RBHcAg_MIR-II_NC-VIII was also validated by histopathological examination of lung tissues. Typical pulmonary inflammations, including inflammatory cell infiltration surrounding the trachea and bronchus, and serous exudation in pulmonary alveoli, occurred in the lung tissues of the mice from the PBS groups. On the contrary, normal lung tissues with only focal mild inflammation were observed in the mice of the vaccine group, which is similar to the histopathological state of the control group (Fig. 5d). In summary, live-virus challenge experiments demonstrated that the designed chimeric RBHcAg_MIR-II_NC-VIII harboring multiple neutralizing epitopes of RSV F protein can protect mice from challenge with RSV A2 virus, which may serve as an antigen candidate for the VLP-based, epitope-focused RSV vaccine design.

## Discussion

The identification of potent anti-RSV nAbs and structural insights into the epitopes targeted by the antibodies enable the design of epitope-focused vaccines against RSV. However, neutralizing epitopes are usually small in size and thus weak in immunogenicity. One of the promising strategies to enhance the immunogenicity is to present the epitopes on the surface of VLP carriers. Many VLPs have been proven to be able to accommodate foreign epitopes without destroying particle assembly, among which HBcAg is a commonly used one. However, previous studies showed that the RSV F protein epitope II displayed on the surface of HBcAg failed to elicit a neutralizing antibody response^23^. In the present study, the HcAgs derived from different animals including WHcAg, GSHcAg, AGSHcAg, BTHcAg and RBHcAg, which are homologous to HBcAg, were used as scaffolds to present epitope II of RSV F protein. Our results demonstrated that these different HcAg-based chimeric VLPs elicited widely varying neutralizing antibody responses, among which the RBHcAg displaying the RSV F protein epitope II is the most immunogenic. Our group has applied RBHcAg as a scaffold for the foreign epitope display, and found that it was highly immunogenic^30^, providing the possible use of RBHcAg-based chimeric VLP construction technology for the display of other epitopes to improve the immune response. In this study, our results demonstrated that RBHcAg can act as an effective VLP carrier to present RSV F epitope II, and its immunogenicity was significantly higher than that of HBcAg as well as most of the other animal-derived HcAgs.

The amino acid sequences of MIR are highly varied among different HcAgs, which may influence the presentation of the inserted epitope conformation. The RBHcAg_MIR-II exhibited higher binding ability than HBcAg_MIR-II to palivizumab (Fig. 2d and Supplementary Table 1), indicating better presentation of the epitope conformation, which may contribute to the higher neutralizing antibody response induced by this chimeric VLP (Fig. 3b and Supplementary Table 3). However, the binding ability of AGSHcAg_MIR-II VLP to palivizumab was comparable to that of HBcAg_MIR-II (Fig. 2d and Supplementary Table 1), but the neutralizing antibody titers induced by AGSHcAg_MIR-II were higher than those of HBcAg_MIR-II, which indicates that the properties of the scaffold may also importantly impact the immunogenicity of the chimeric VLP antigen (Fig. 3b and Supplementary Table 3). Another advantage of applying animal-derived HcAgs as epitope-presenting scaffolds is that they may be more effective than HBcAg in human use owing to the absence of pre-existing antibodies.

Using the most immunogenic RBHcAg as the carrier, we then designed the chimeric VLPs displaying multiple neutralizing epitopes from RSV F protein. Our results show that besides epitope II presented on the MIR, the additional insertion of epitope VIII at the N- and C-termini can further improve the neutralizing antibody level. The incorporation of multiple epitopic regions into the chimeric VLP may facilitate the generation of multiple types of neutralizing antibodies and increase the immunogenicity of the antigen. Live-virus challenge experiments demonstrated that the presentation of several small immunogenic epitopes on the surface of RBHcAg induced substantial protection in mice against the infection of RSV. The RBHcAg-based multiple-epitope-display VLPs may serve as the potential starting point for the development of epitope-focused vaccines against RSV.

Many studies have revealed that the VED primed by the inactivated RSV vaccine is attributed to the imbalanced Th1/Th2 immunity and non-neutralizing antibodies^37,38,44^. Our experimental results show that the designed epitope-display VLPs plus MF59-like adjuvant elicited a more balanced Th1/Th2 immune response compared with the inactivated vaccine, which implies the potential safety of the hybrid VLPs for possible vaccine development. In our study, the designed VLPs were formulated with MF59-like adjuvant for mouse immunization. MF59 has been proven to elicit more Th1-biased immune responses than aluminum salt adjuvant^45^. The balanced Th1/Th2 immune response of the chimeric VLPs may be attributed to the use of MF59-like adjuvant.

Recently, two vaccines, i.e., Arexvy made by GSK and Abrysvo by Pfizer, were approved by the U.S. Food and Drug Administration (FDA) to protect against RSV. Both vaccines used the RSV F protein stabilized in its prefusion conformation as the antigen^11,46^. Our study adopted another strategy for possible RSV vaccine design, in which the potent neutralizing epitopes derived from RSV F protein were grafted onto the surface of the virus-like particle (VLP) formed by the hepadnavirus core proteins. One of the advantages of this epitope-focused design strategy is to retain the neutralizing epitopes and delete the non-neutralizing regions of the antigen, which enables the induction of neutralizing antibody response targeting the specific epitopes and elimination of the non-neutralizing immunity. The RBHcAg-based epitope-display scheme developed in our study may serve as an alternative possible approach for the development of RSV vaccines.

## Methods

### Construction, recombinant expression and purification of the HcAg-based chimeric VLPs

The sequences of hepadnavirus core proteins (HcAgs) derived from different species, including human (HBcAg), woodchuck (WHcAg), ground squirrel (GSHcAg), arctic ground squirrel (AGSHcAg), bat (BTHcAg) and roundleaf bat (RBHcAg), were downloaded from the Genebank or Swiss-Prot with the accession Numbers QIP67541.1, NC_004107, P0C6J1.1, Q64897.1, AIW47294.1 and YP_009045994.1, respectively. The neutralizing epitopes II and VIII were taken from the F protein of the RSV A subgroup with the amino acids 254–277 and 163–181, respectively. For the construction of the single-epitope-display VLPs, two short exogenous linkers “GILE” and “L” were added^24^, respectively, to the two ends of epitope II, which was then inserted into the MIR region at the site 78/79 of different HcAgs. For the construction of RBHcAg-based multiple-epitope-presenting VLPs, another epitope II without exogenous linkers or epitope VIII was further fused to the N-terminus or C-terminus or both N- and C-termini of RBHcAg. The constructed chimeric protein sequences were codon-optimized and cloned into the shuttle plasmid, which was then transformed into the *Hansenula polymorpha* expression system, developed by our laboratory, for the recombinant expression of the proteins as described in our previous papers^33,34^. After 5 days of cultivation, the yeast cells were collected and crushed to obtain the supernatant. Then, the recombinant chimeric VLPs were purified by using gel-filtration chromatography with the Sephacryl S500-HR medium (General Electric Company).

### SDS-PAGE and western blotting

The expression of the chimeric proteins was detected by using SDS-PAGE and Western-Blot. In SDS-PAGE, the purified protein sample was prepared to the concentration of 200 μg/ml. 30 μL of the sample were separated in the 12% SDS-PAGE gel and stained with coomassie brilliant blue staining. In Wester-Blot, the SDS-PAGE gels were transferred to PVDF membranes and blocked by TBST with 5% nonfat dry milk. The expression of target protein was recognized by palivizumab (MedImmune Inc., Synagis®), at a concentration of 2.5 μg/ml, combined with HRP-conjugated goat anti-human IgG antibody (purchased from ZSGB-BIO, Cat number: ZB-2304) at a 1:4000 dilution. Color was developed with 3,3’-diaminobenzidine (DAB), and bands were visualized and recorded. The uncropped and unprocessed gel images and blots are provided in Supplementary Fig. 1. All blots or gels derive from the same experiment and they were processed in parallel.

### TEM observations

The morphology of the recombinant chimeric VLPs was observed by transmission electron microscope (TEM). The protein sample was dropped onto the carbon-coated copper grids and adsorbed for 5 min. The surplus sample was absorbed using filter paper. The sample on copper grids was negatively stained with 2% phosphotungstic acid solution (pH 7.0) for 1 min. After removing the excess solution with absorbent paper, the copper grids were dried at room temperature. Then, the morphology of the VLP sample was observed using the Hitachi JEM-1400 TEM.

### ELISA assay

The binding activity of the designed chimeric VLPs to the palivizumab was detected by using the enzyme-linked immunosorbent assay (ELISA). The binding strength of the native RSV F protein to palivizumab was also monitored as a control. The protein sample was firstly diluted to the concentration of 1 μg/ml, followed by two-fold serial dilutions. The diluted sample was coated onto the wells of the plate with 100 μL per well at 4 °C for 8 h. After washing three times using phosphate-buffered saline with Tween 20 (PBST), the plate was blocked by SuperBlock™ blocking buffer with 150 μL per well at 37 °C for 3 h, and washed three times with PBST. Then, palivizumab was diluted to 0.5 μg/mL, and added to the wells with 100 μL per well. The plate was incubated at 37 °C for 1.5 h and washed with PBST for three times. Subsequently, HRP-conjugated goat anti-human IgG antibody was diluted 1:10000, and added to the plate with 100 μL per well. After incubation at 37 °C for 1 h, the plate was washed with PBST for three times. Then, 50 µL tetramethylbenzidine (TMB) and 50 µL hydrogen peroxide solutions were added to each of the wells to develop color at room temperature for 5 min. The color reaction was terminated by adding 50 µL 0.2 M sulfuric acidic solution to each well. The intensity of the absorbance, also known as optical density (OD), at the wavelength of 450 nm was read. After subtracting the background absorbance at 630 nm, the OD_450nm/630nm_ value was obtained to evaluate the binding strength of the protein sample with palivizumab. Three wells were set for each sample at each concentration, and the average value of the three repeated measurements was reported.

To assess the relative binding affinities of the chimeric VLPs in comparison to the native RSV F protein, a competitive palivizumab-binding assay was also carried out by using ELISA. In this assay, RSV F protein was diluted to 0.5 μg/mL and coated onto the well with 100 μL per well, followed by blocking with the SuperBlock™ blocking buffer. The recombinant chimeric VLP sample was diluted to 20 μg/ml and then subjected to two-fold serial dilutions. 50 μL of the diluted sample and an equal volume of palivizumab with 0.1 μg/mL were added to the well. The wells added by 50 μL dilution buffer mixed with an equal volume of 0.1 μg/mL palivizumab were set as a positive control. Then, similar to the above procedure, the HRP-conjugated goat anti-human IgG antibody at a 1:10000 dilution was used as the secondary antibody for color development. The average OD_450nm/630nm_ value of triplicate measurements was obtained. The RSV F-palivizumab binding inhibition rate was calculated as $\left(1-{{{\rm{OD}}}}_{450\,{{\rm{nm}}}/630\,{{\rm{nm}}}}^{{{\rm{VLP}}}}/{{{\rm{OD}}}}_{450\,{{\rm{nm}}}/630\,{{\rm{nm}}}}^{{{\rm{control}}}}\right)\times 100 \%$, where ${{{\rm{OD}}}}_{450\,{{\rm{nm}}}/630\,{{\rm{nm}}}}^{{{\rm{VLP}}}}$ is the OD_450nm/630nm_ value of the wells containing the VLP sample, and ${{{\rm{OD}}}}_{450\,{{\rm{nm}}}/630\,{{\rm{nm}}}}^{{{\rm{control}}}}$ is the value of the positive control wells.

For IgG1 and IgG2a antibody detections in the serum of the immunized mice by ELISA, RSV F protein was diluted to 0.1 μg/ml, which was then coated onto the plate wells and blocked with SuperBlock™ buffer. The serum sample from the immunized mice was three-fold serially diluted and added to the wells for incubation at 37 °C for 1.5 h. The HRP-labeled goat anti-mouse IgG1 (purchased from Southern Biotech, Cat number: 1070-05) at a 1:10000 dilution and HRP-labeled goat anti-mouse IgG2a (purchased from Southern Biotech, Cat number: 1080-05) at a 1:10000 dilution were respectively used as the secondary antibodies for the corresponding IgG sub-class detections.

### Mouse immunization

All animal experiments have been approved by the Institutional Animal Care and Use Committee (IACUC) of the National Vaccine and Serum Institute (NVSI) of China, and conducted under the regulations for the administration of affairs concerning experimental animals of China (2017). The immunogenicity of the designed chimeric VLPs was evaluated in mice. The chimeric VLPs were complexed with home-made MF59-like adjuvant, and each dose contains 30 μg antigen (150 μL) and 150 μL adjuvant. The MF59-like adjuvant, containing 3.9% squalene, 0.47% polysorbate 80 and 0.47% sorbitan trioleate, was prepared using the dynamic high-pressure microfluidization with the pressure of 10000 pounds per square inch by 5 cycles.

To evaluate the immunogenicity of the designed chimeric VLPs that use different species of HcAg as the scaffolds to display epitope II of the RSV F protein, 8 female BALB/c mice (purchased from Charles River), aged 6–8 weeks and weighting 18–22 g, were vaccinated for each VLP by three doses with intervals of two weeks. Another group of mice administered by adjuvant with the same regimen was used as a control. Two weeks after the third immunization, blood samples were collected from the eye vein of the immunized mice, and serum was separated from the blood sample for antibody detections.

To evaluate the immunogenicity of the designed chimeric VLPs harboring multiple RSV F neutralizing epitopes, 8 female BALB/c mice were immunized for each VLP with the same regimen as described above. Another group of mice was administered with the RBHcAg_MIR-II that harbors a single RSV F epitope II as a comparison. Two weeks after completion of the immunization, serum samples were collected from the eye vein of the mice.

To evaluate the cellular immunity elicited by RBHcAg_MIR-II_NC-VIII and compared with that induced by the formalin-inactivated RSV (FI-RSV). Mice (*n* = 8 per group) were immunized with adjuvanted RBHcAg_MIR-II_NC-VIII or FI-RSV by the same regimen as described above. Each dose of FI-RSV contained 2.5 × 10^5^ TCID_50_ RSV A2 virus and 320 μg aluminum hydroxide adjuvant. Another group administered with phosphate buffer saline (PBS) as a control. Two weeks post-immunization, serum samples were collected from the eye vein of the mice for IgG1 and IgG2a antibody detections. In each group, four mice were randomly selected and sacrificed by cervical dislocation after anesthesia with an intraperitoneal injection of 0.35% pentobarbital sodium (40–50 mg/kg). The spleens were isolated from the mice for cytokine secretion level detections.

### RSV virus culture

RSV A subgroup Long strain (ATCC VR-26) and A2 strain (ATCC VR-1540) were propagated in HEp-2 cells (ATCC CCL-23). The cells were inoculated with virus at a 0.02 multiplicity of infection (MOI), which was then placed in a CO_2_ incubator at 37 °C for adsorption two hours. Then, the DMEM culture medium containing 2% FBS and 1% penicillin-streptomycin (double antibody) was added, and the virus was cultured at 37 °C with 5% CO_2_. The cells were harvested every day from 3 to 7 days, respectively, and then disrupted by freezing and thawing at −80 °C. The supernatant was collected by centrifugation. The virus titer in the harvested supernatant was measured, based on which, the optimal virus harvesting time was determined. Large-scale virus cultivation was conducted under the determined optimal conditions. For the inactivated virus used in our study, the RSV A2 strain was treated with formalin according to the Lot 100 method^4,47^. Briefly, The RSV-containing cell culture supernatant was inactivated by formalin with a concentration of 1:4000 at 37 °C for 72 h. After concentrating 25-fold by ultracentrifugation, the virus was absorbed to aluminum hydroxide adjuvant at the room temperature overnight. Then, it was pelleted by centrifugation and resuspended to 1/4^th^ volume to produce the final vaccine.

### Live-virus neutralization assay

The neutralizing antibody level in the serum of the immunized mice was evaluated by using the live-virus neutralization assay. HEp-2 cells were seeded at a density of 1.0–2.0 × 10^5 ^ml^−1^ in the 96-well plate with 100 μl per well, and incubated under 5% CO_2_ at 37 °C for 16~24 h. The serum sample was two-fold serially diluted and mixed with an equal volume of 1200 TCID_50_/ml (50 μl per well) RSV Long strain, which was incubated at 4 °C for 1 h. Then, the serum-virus mixture was added to the cell culture plate with 100 μl per well and incubated under 5% CO_2_ at 37 °C for 3 days. Another four wells added by the virus without serum were set as positive controls, and four wells by only the cell culture medium as negative controls. Subsequently, the cells were fixed in 4% formaldehyde, permeabilized with 0.5% Triton X-100, and incubated with 5% bovine serum albumin (BSA) for 90 min. The cells were then stained with anti-RSV N protein mouse monoclonal antibody (purchased from Millipore, Cat number: MAB858-3) at a dilution of 1:1000 and Alexa Fluor® 488-labeled goat anti-mouse IgG antibody (purchased from ZSGB-BIO, Cat number: ZF-0512) at a dilution of 1:500. Then, the green fluorescence signals in the HEp-2 cells were observed under the inverted fluorescence microscope, and the neutralizing antibody titer was determined as the reciprocal of the maximum serum dilution that 50% inhibits the viral infection. All the assays were performed in duplicate wells, and the average value was reported.

### Cytokine secretion level detections

The spleens were separated from the immunized mice after anesthesia and sacrifice as described above, and the splenic lymphocytes were isolated using the red blood cell lysis method, which were then stimulated using the live RSV A2 virus under the co-stimulation of the anti-mouse CD28 antibody (2.5 μg/ml, Thermo/Invitrogen, Cat number: 16-0281-85) and the anti-mouse CD49d antibody (2.5 μg/ml, Thermo/Invitrogen, Cat number: 16-0492-85). After cultivation under 5% CO_2_ at 37 °C for 72 h, the interleukin (IL)-4 and interferon Gamma (IFN-γ) secretion levels in the supernatant were detected using the MILLIPLEX Mouse Cytokine/Chemokine Magnetic Bead Panel (MERCK, MCYTOMAG-70K).

### Live RSV virus challenge in mice

In order to test the protective efficacy of the designed multiple-epitope-display RBHcAg_MIR-II_NC-VIII VLP, mice were immunized and challenged by the live RSV A2 strain. Two groups (8 mice per group) of 6 to 8-weeks-old female BALB/c mice that are weighting 18–22 g were intraperitoneally immunized by three doses of the recombinant RBHcAg_MIR-II_NC-VIII VLP in formulation with MF59-like adjuvant (vaccine group) or by three doses of PBS (PBS group), with intervals of two weeks. Two weeks after the third vaccination, mice were anesthetized with an intraperitoneal injection of 0.35% pentobarbital sodium (40–50 mg/kg), and challenged with 3 × 10^5^ TCID_50_ live RSV A2 virus via intranasal route. Another group of mice immunized and challenged by PBS with the same regimen was used as a control (control group). During the challenge experiment, the body weights of the mice were measured and recorded. Five days post-challenge, mice were sacrificed by cervical dislocation after anesthesia by 0.35% pentobarbital sodium, and the lung tissues were collected. The viral RNAs were extracted from the lung tissues by TRIzol (Thermo Fisher Scientific, Cat number: 15596026), according to the manufacturer’s instructions. Reverse transcription real-time PCR was performed using the AgPath-ID One-step RT-PCR Kit (Thermo Fisher Scientific, Cat number: AM1005). The expression levels of RSV N RNA in the cultured cells were calculated by the comparative cycle threshold (Ct) method using the expression level of the GAPDH gene as the calibrator, and the results were reported as the fold change of the viral loads in comparison to the mean value of the PBS group^48^. For histopathological examinations, the lung tissue samples were stained with hematoxylin and eosin (H&E), and the histopathological changes in the lung tissues were examined.

### Quantification and statistical analysis

SAS 9.4 software was used for statistical analysis. Data was first tested for normal distribution using the Shapiro-Wilk test. For data with normal distribution, one-way ANOVA followed by the LSD t-test was used for multiple-group comparison. If the data did not follow a normal distribution, the Kruskal–Wallis test was used for multiple comparisons. A two-sided *p* < 0.05 was considered statistically significant. *p < 0.05, **p < 0.01, ***p < 0.001, ****p < 0.0001, ns not significant.

### Reporting summary

Further information on research design is available in the Nature Research Reporting Summary linked to this article.

### Supplementary information

Supplementary material

REPORTING SUMMARY

## Data Availability

All data supporting the findings of this study are available in the paper and the Supplementary Material. Any additional information is available from the corresponding author upon request.
