# Supplementary material for: Design of hepadnavirus core protein-based chimeric virus-like particles carrying epitopes from respiratory syncytial virus
Source: NPJ Vaccines. 2024 Mar 19;9:62. doi: 10.1038/s41541-024-00855-7 (PMC10951352; doi:10.1038/s41541-024-00855-7)
Supplement: Supplementary file 1 — Supplementary material [file 41541_2024_855_MOESM1_ESM.pdf]

Supplementary Material

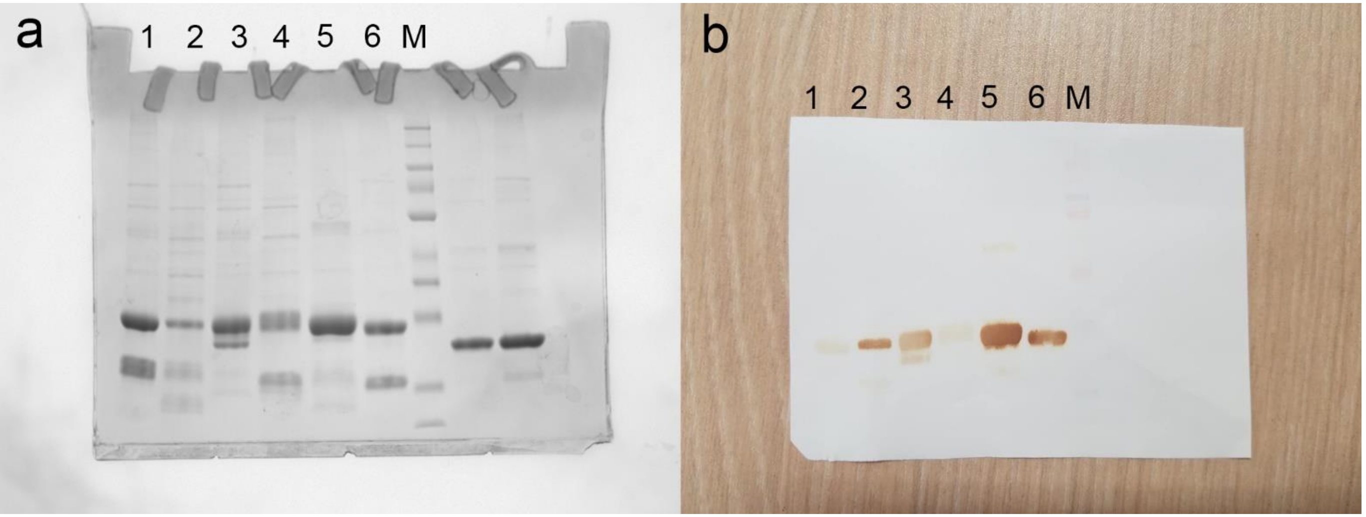

Supplementary Figure 1. The uncropped and unprocessed gel images and blots. a The uncropped and unprocessed SDS-PAGE image for Figure 2a in the main text. **b** The uncropped and unprocessed Western-blot for Figure 2b in the main text. Lane 1: BTHcAg_MIR-II, Lane 2: AGSHcAg_MIR-II, Lane 3: WHcAg_MIR-II, Lane 4: HBcAg_MIR-II, Lane 5: GSHcAg_MIR-II, Lane 6: RBHcAg_MIR-II, Lane M: Protein molecular weight marker.

Supplementary Table 1. $OD_{450/630nm}$ values for the binding of the designed chimeric VLPs at different concentrations to palivizumab detected by ELISA. Three repeated measurements were performed.

VLP concentration （ng/ml)	RBHcAg_MIR-II			BTHcAg_MIR-II			AGSHcAg_MIR-II			GSHcAg_MIR-II			WHcAg_MIR-II			HBcAg_MIR-II			Native F		
1000	2.179	2.22	2.276	2.137	2.143	2.217	2.112	2.11	2.12	2.261	2.209	2.274	2.197	2.146	2.149	2.183	2.112	2.14	2.076	2.199	2.029
500	2.106	2.109	2.118	2.141	2.128	2.093	2.09	2.058	1.957	2.152	2.23	2.109	2.1	2.154	2.092	1.94	1.902	1.957	1.861	1.976	1.787
250	2.039	1.952	2.104	2.034	1.972	2.004	1.924	1.736	1.781	2.027	2.026	2.072	1.969	1.981	1.984	1.692	1.652	1.628	1.498	1.571	1.282
125	1.84	1.867	1.804	1.926	1.929	1.884	1.516	1.493	1.462	2.004	1.923	1.983	1.815	1.762	1.737	1.531	1.471	1.388	1.135	1.085	0.879
62.5	1.535	1.472	1.567	1.649	1.637	1.614	1.051	0.957	1.004	1.645	1.612	1.606	1.41	1.358	1.331	1.101	0.857	0.898	0.685	0.599	0.437
31.25	1.188	1.008	1.068	1.343	1.21	1.226	0.624	0.586	0.597	1.31	1.266	1.175	0.978	1.106	0.928	0.64	0.595	0.524	0.399	0.35	0.254
15.625	0.761	0.699	0.661	0.872	0.797	0.749	0.321	0.29	0.275	0.806	0.782	0.758	0.569	0.513	0.492	0.338	0.345	0.282	0.19	0.199	0.127
7.8125	0.369	0.348	0.333	0.492	0.435	0.403	0.151	0.129	0.128	0.47	0.414	0.382	0.302	0.262	0.268	0.181	0.147	0.14	0.111	0.102	0.079
3.90625	0.208	0.196	0.156	0.26	0.248	0.192	0.091	0.081	0.065	0.236	0.229	0.193	0.159	0.156	0.146	0.092	0.081	0.07	0.059	0.049	0.038
1.953125	0.106	0.098	0.072	0.137	0.129	0.097	0.042	0.045	0.035	0.121	0.132	0.099	0.08	0.07	0.073	0.05	0.042	0.036	0.033	0.03	0.022
0.9765625	0.05	0.064	0.041	0.066	0.069	0.05	0.022	0.029	0.02	0.062	0.077	0.051	0.043	0.045	0.039	0.026	0.029	0.026	0.02	0.021	0.016

Supplementary Table 2. Data for the competitive palivizumab-binding abilities of the designed chimeric VLPs in contrast with the native RSV F protein tested using ELISA (%inhibition). Three repeated measurements were performed.

VLP concentration （ng/well)	RBHcAg_MIR-II			BTHcAg_MIR-II			AGSHcAg_MIR-II			GSHcAg_MIR-II			WHcAg_MIR-II			HBcAg_MIR-II		
1000	91.532	92.201	90.949	92.808	94.919	94.127	76.323	77.349	74.739	87.953	88.653	80.185	70.274	83.635	82.408	18.948	16.257	13.861
500	91.679	90.354	92.228	89.291	92.276	90.756	70.404	70.699	67.223	83.209	84.974	78.043	68.678	76.097	69.189	6.129	7.893	8.550
250	90.994	87.943	87.752	85.083	87.652	86.786	60.298	62.361	59.603	77.719	78.238	67.868	55.611	55.391	56.806	5.873	5.123	3.452
125	87.518	87.122	85.047	81.140	82.571	80.750	50.914	50.072	45.355	60.821	66.995	56.378	39.052	36.116	36.855	-0.715	0.680	2.337
62.5	84.043	84.813	82.243	71.444	72.967	70.799	34.360	30.651	19.468	52.399	54.249	40.896	15.960	18.941	20.197	3.320	0.784	-1.859
31.25	82.085	81.375	74.816	48.002	57.622	54.377	23.917	21.976	15.292	28.571	31.088	25.122	9.426	5.964	10.123	-0.562	-1.882	-1.221
15.625	65.884	63.212	64.142	33.777	43.852	34.095	3.609	9.060	-4.697	16.951	18.135	7.157	-1.097	4.723	8.501	-0.409	-0.261	-4.886
7.8125	51.346	39.610	47.860	20.671	25.762	13.812	5.438	9.349	-5.793	4.797	4.456	3.262	-0.449	-0.620	3.931	2.809	-3.293	-3.877
3.90625	21.586	14.007	18.642	3.889	9.350	4.785	2.984	8.771	-5.115	4.371	4.404	-0.097	0.449	4.771	10.074	1.992	0.261	-2.602
1.953125	10.328	4.002	8.165	7.405	2.896	-3.861	-0.674	10.458	-4.175	2.612	6.321	-2.240	1.297	5.677	6.486	0.613	-0.418	-5.151
0.9765625	8.811	3.438	8.116	3.197	4.167	-0.109	7.748	11.229	-3.288	2.878	5.959	3.797	4.439	1.002	6.241	3.626	0.314	-2.655

Supplementary Table 3. Individual data of the live-virus neutralizing antibody titers in the serum of the mice immunized with different chimeric VLPs that display the RSV F epitope II.

Design schemes	Mouse 1	Mouse 2	Mouse 3	Mouse 4	Mouse 5	Mouse 6	Mouse 7	Mouse 8	GMT
RBHcAg_MIR-II	1024	2048	128	64	128	64	512	64	215.2695
BTHcAg_MIR-II	16	16	16	16	16	32	32	1024	32
AGSHcAg_MIR-II	32	32	32	32	128	128	32	4096	82.99773
GSHcAg_MIR-II	64	16	32	64	512	32	32	64	53.81737
WHcAg_MIR-II	32	16	64	16	64	256	64	32	45.25483
HBcAg_MIR-II	16	32	16	64	16	32	64	16	26.90869
Adjuvant	16	8	16	8	8	8	16	8	10.37472

Note: The quantification limit of the live-virus neutralization assay was 16, and the titer value below the limit of detection (LOD) was set to 8.

Supplementary Table 4. Individual data of live-virus neutralizing antibody titers in the mice elicited by different chimeric VLPs harboring multiple RSV F neutralizing epitopes.

Design schemes	Mouse 1	Mouse 2	Mouse 3	Mouse 4	Mouse 5	Mouse 6	Mouse 7	Mouse 8	GMT
RBHcAg_MIR-II_NC-VIII	256	512	8192	2048	1024	256	2048	128	789.6119
RBHcAg_MIR-II_NC-II	1024	2048	2048	4096	1024	256	4096	128	1116.68
RBHcAg_MIR-II_C-II	128	64	1024	4096	256	512	512	128	362.0387
RBHcAg_MIR-II_N-II	16	16	32	32	16	16	32	32	22.62742
RBHcAg_MIR-II	1024	2048	128	64	128	64	512	64	215.2695

Note: The quantification limit of the live-virus neutralization assay was 16.

Supplementary Table 5. Individual data for the IgG1 and IgG2a antibody titers induced by RBHcAg_MIR-II_NC-VIII in comparison to those induced by FI-RSV.

Design schemes	IgG2a								IgG1							
	Mouse 1	Mouse 2	Mouse 3	Mouse 4	Mouse 5	Mouse 6	Mouse 7	Mouse 8	Mouse 1	Mouse 2	Mouse 3	Mouse 4	Mouse 5	Mouse 6	Mouse 7	Mouse 8
RBHcAg_MIR-II_NC-VIII	2700	2700	900	900	300	900	2700	8100	8100	900	2700	900	72900	8100	2700	2700
FI-RSV	2700	24300	300	50	300	100	/	/	24300	2700	8100	24300	8100	24300	/	/
PBS	50	50	50	50	50	50	50	50	50	50	50	50	50	50	50	50

Note: "/" means that the mouse died. The quantification limit of the assay was 100, and the titer value below the limit of detection (LOD) was set to 50.

Supplementary Table 6. Individual data for the IL-4 secretion levels in the splenic lymphocytes of the mice immunized with RBHcAg_MIR-II_NC-VIII in comparison to those immunized with FI-RSV (pg/10^6 cells).

	Mouse 1	Mouse 2	Mouse 3	Mouse 4
RBHcAg_MIR-II_NC-VIII	51.00671	38.74233	21.66667	59.55056
FI-RSV	54.81865	0	0	69.0625
PBS	4.603175	4.987179	6.3	1.530287

Supplementary Table 7. Individual data for the IFN-γ secretion levels in the splenic lymphocytes of the mice immunized with RBHcAg_MIR-II_NC-VIII in comparison to those immunized with FI-RSV (pg/10^6 cells).

	Mouse 1	Mouse 2	Mouse 3	Mouse 4
RBHcAg_MIR-II_NC-VIII	677.5503	255.8896	170.1366	720.2247
FI-RSV	75.12953	0	58.21826	0
PBS	11.26984	5.948718	19.03333	2.274176

Supplementary Table 8. Individual data for the body weight changes of the mice from different groups after live-virus challenge. The unit of body weight is g.

		Mouse 1	Mouse 2	Mouse 3	Mouse 4	Mouse 5	Mouse 6	Mouse 7	Mouse 8
Vaccine group	Day 0	26.94	24.74	23.52	20.35	25.14	24.14	20.72	23.69
	Day 1	20.26	23.11	25.69	19.8	26.01	23.6	23.43	24.56
	Day 2	24.07	22.7	19.91	19.87	25.48	22.68	25.56	24.08
	Day 3	21.59	24.65	20.68	24.01	25.37	25.91	26.11	23.84
	Day 4	24.12	20.3	21.41	25.96	24.84	25.6	23.82	25.88
	Day 5	20.77	25	21.69	26.9	23.95	26.51	25.35	26.28
PBS group	Day 0	23.25	23.42	23.77	23.52	23.34	22.73	24.31	24.43
	Day 1	22.89	21.3	22.02	23.75	21.91	21.91	21.12	/
	Day 2	22.71	23.7	22.24	21.55	23.04	22.13	21.54	/
	Day 3	23.21	24.56	23.59	21.93	21.93	24.38	22.23	/
	Day 4	23.99	24.2	23.24	22.3	21.98	23.84	22.25	/
	Day 5	23.2	24	23.93	22.18	22	22.68	23.99	/
Control group	Day 0	24.32	24.26	25.17	23.35				
	Day 1	24.29	23.67	23.16	23.32				
	Day 2	23.17	24.03	23.95	23.79				
	Day 3	24.74	24.32	24.38	24.23				
	Day 4	24.46	24.69	23.79	23.75				
	Day 5	23.83	24.2	24.96	24.75				

Note: "/" means that the mouse died during the live-virus challenge experiment.

Supplementary Table 9. Individual data for the viral loads in the lung tissues of the mice from different groups. The viral loads were calculated by the comparative cycle threshold (Ct) method using the expression level of the GAPDH gene as the calibrator. Detection results were reported as the fold changes of the viral loads in comparison to the mean value of the vaccine group.

	Mouse 1	Mouse 2	Mouse 3	Mouse 4	Mouse 5
Vaccine group	0.11682	0.00020	0.05150	0.00022	0.01032
PBS group	1.19425	1.20948	1.14131	0.45496	/
Control	0.00052	0.00042	0.00044	/	/

Note: "/" means no mouse.
